# Development of an immunogenic cell death prognostic signature for predicting clinical outcome and immune infiltration characterization in stomach adenocarcinoma

**DOI:** 10.18632/aging.205132

**Published:** 2023-10-19

**Authors:** Ye Liu, Lijia Zhang, Xue Lei, Xinyu Yin, Songjiang Liu

**Affiliations:** 1 Department of Intensive Care Unit, First Affiliated Hospital, Heilongjiang University of Chinese Medicine, Harbin 150040, Heilongjiang Province, China; 2 Ethics Committee Office, First Affiliated Hospital, Heilongjiang University of Chinese Medicine, Harbin 150040, Heilongjiang Province, China; 3 Department of Clinical Specialty of Integrated Traditional Chinese and Western Medicine, Graduate School, Heilongjiang University of Chinese Medicine, Harbin 150040, Heilongjiang Province, China; 4 Department of Oncology, First Affiliated Hospital, Heilongjiang University of Chinese Medicine, Harbin 150040, Heilongjiang Province, China

**Keywords:** immunogenic cell death, molecular subtype, immune infiltration, prognostic value, tumor mutation burden

## Abstract

Stomach adenocarcinoma (STAD) is a common gastric histological cancer type with a high mortality rate. Immunogenic cell death (ICD) plays a key factor during carcinogenesis progress, whereas the prognostic value and role of ICD-related genes (ICDRGs) in STAD remain unclear. The MSigDB database collecting ICDRGs were selected by univariate Cox regression analysis and LASSO algorithm to establish a novel risk model. The Kaplan-Meier survival analysis indicated a significant difference of OS rate of patients by risk score stratification. ESTIMATE, CIBERSORT, and single sample gene set enrichment analysis (ssGSEA) algorithms were conducted to estimate the immune infiltration landscape by risk stratification. Subgroup analysis and tumor mutation burden analysis were also analyzed to identify characteristics between groups. Differences in therapeutic responsiveness to chemotherapeutic drugs and targeted drugs were also analyzed between high-risk group and low-risk group. The impact of one ICDRG, GPX1, on the proliferation, migration and invasiveness of was confirmed by *in vitro* experiments in GC cells to test the reliability of bioinformatics results. This study gives evidence of the involvement of ICD process in STAD and provides a new perspective for further accurate assessment of prognosis and therapeutic efficacy in STAD patients.

Stomach adenocarcinoma (STAD) is a common gastric histological cancer type with a high mortality rate. Immunogenic cell death (ICD) plays a key factor during carcinogenesis progress, whereas the prognostic value and role of ICD-related genes (ICDRGs) in STAD remains unclear. The MSigDB database collected ICDRGs were selected by univariate Cox regression analysis and LASSO algorithm to establish a novel risk model. The Kaplan-Meier survival analysis indicated a significant difference of OS rate of patients by risk score stratification. ESTIMATE, CIBERSORT, and single sample gene set enrichment analysis (ssGSEA) algorithms were conducted to estimate the immune infiltration landscape by risk stratification. Subgroup analysis and tumor mutation burden analysis were also analyzed to identify characteristics between groups. Differences in therapeutic responsiveness to chemotherapeutic drugs and targeted drugs were also analyzed between high-risk group and low-risk group. The impact of one ICDRG, GPX1, on the proliferation, migration and invasiveness of was confirmed by *in vitro* experiments in GC cells to test the reliability of bioinformatics results. This study gives evidence of the involvement of ICD process in STAD and provides a new perspective for further accurate assessment of prognosis and therapeutic efficacy in STAD patients.

## INTRODUCTION

Gastric cancer (GC) is one of the most common cancer types worldwide. As a highly aggressive malignancy, more than one million GC new cases and nearly 760,000 deaths were reported worldwide by 2020 [1]. Stomach adenocarcinoma (STAD) is a common gastric histological cancer type with a high mortality rate [2]. Since most STAD patients are found at late stages, systemic therapy based on novel therapeutic approaches and targets remains a top priority [3]. Therefore, better risk assessment of STAD patients will help in the selection of treatment options.

As a type of regulated cell death, immunogenic cell death (ICD) is usually driven by stress and accompanied by the active secretion or passive release of a large number of damages related molecular patterns (DAMPs), such as adenosine triphosphate (ATP), heat shock protein (HSP), calreticulin (CRT) and high mobility group box 1 (HMGB1) [4]. During ICD process, dying cells identified by expressing of pattern recognition receptors (PRRs) release “eat me” or “find me” signals [5]. These recognized signals stimulate the recruitment and activation of various immune cell subtypes, including neutrophils and macrophages, resulting in an effective immune response [6].

The anti-tumor immune response induced by tumor programmed cell death can enhance the therapeutic effect, however, the role of tumor microenvironment (TME) in STAD has not been fully reported [7]. It has been reported that the prognostic value of TME in patients with STAD and its correlation with immunotherapy sensitivity [8]. Possible factors affecting prognosis through the regulation of TME include m6A modification, histone lysine demethylases, nucleotide metabolism and neuroendocrine regulation, etc. [9–12]. Components such as monocytes, resting mast cells, and M2 macrophages were also reported to be associated with the expression levels of specific genes that influence prognosis [13]. In addition, it has been reported that the incidence of prognostic mutations such as TP53 is related to the composition of the immune microenvironment [14]. As an immune-related process, ICD also plays a role through the regulation of TME. ICD process effectively activates immune responses and triggers tumor-specific adaptive immunity by identifying DAMPs released by dying cells in the TME [15, 16]. A growing number of preclinical and clinical evidence suggests that many successful antitumor therapies have benefited from the effective induction of ICD in tumor cells [17, 18]. Although it has been reported that ICD may be involved in the course of oxaliplatin combined with immune checkpoint inhibitors in GC patients, the effect of ICD on the therapeutic effect of GC and STAD and whether TME regulation is involved in this process are unclear [19].

In this study, we systematically investigated the relationship between ICD-related genes (ICDRGs) and clinicopathological features of STAD patients based on TCGA data. A risk model of STAD patients based on ICDRGs was subsequently constructed and its ability to predict the prognosis was verified. We further comprehensively analyzed the immune microenvironment of STAD patients and explored the impact of risk stratification on immune response and drug sensitivity treatment. The impact of one ICDRG, GPX1, on the proliferation, migration and invasiveness of GC cells was confirmed by *in vitro* experiments to test the reliability of bioinformatics results. This study gives evidence of the involvement of ICD process in STAD and provides a new perspective for further accurate assessment of prognosis and therapeutic efficacy in STAD patients.

## MATERIALS AND METHODS

### Data collection

We selected 34 ICDRGs for our study based on a previous study [20] (Supplementary Table 1). The RNA sequencing (RNA-seq) data, corresponding clinicopathological data, somatic mutation, and copy number variation (CNV) files of gastric cancer (GC) were obtained from The Cancer Genome Atlas (TCGA) database and the Gene Expression Omnibus (GEO) database. After removing cases without survival data (371 from TCGA and 433 from GSE84437), a total of 804 GC samples were enrolled. To eliminate the batch effect of the combined dataset, we utilized the “SVA” and simplify packages for background modification and quantitative normalization.

### Identification of differentially expressed ICDRGs

In both normal and tumor tissues for GC, the Wilcoxon test was employed to conduct a comparative analysis of the differential expression of ICDRGs. Furthermore, a protein-protein interaction analysis was applied by using the STRING database (https://string-db.org/) to investigate the plausible association of ICDRGs.

### Somatic mutation and CNV estimation of ICDRGs

We utilized the R package “Maftools” to investigate the somatic landscape of ICDRGs and generate waterfall plots to summarize the status of mutant genes. The GISTIC algorithm with a q-value threshold of <0.05 was employed to detect the CNV of amplification and deletion in all samples. To investigate the chromosomal position of ICDRGs, we used the R package “RCircos”. Moreover, we compared the percentage numbers of microsatellite stability (MSS), low microsatellite instability (MSI-L) and high microsatellite instability (MSI-H) across different ICDRG score groups.

### Unsupervised consensus clustering analysis

To functionally characterize the molecular subtypes of ICDRGs in GC, we conducted an unsupervised consensus clustering analysis using the “ConcensusClusterPlus” R package. This was based on the 33 ICDRGs expression profiles, with 1,000 iterations and a sampling of 80% of the data, to achieve reliable results in each iteration. GC patients were grouped into clusters A, B, and C using principal component analysis (PCA), and overall survival (OS) for each ICDRGs cluster was assessed using the R package “survival”. To further explore the biological pathway of ICDRGs subtype in GC patients, we used the “GSVA” R package for getting gene set variation analysis (GSVA). The “ESTIMATE” algorithm was also used to calculate the tumor purity and stromal, immune and ESTIMATE scores for each ICDRG cluster subgroup. We also conducted single-sample gene set enrichment analysis (ssGSEA) to assess the 23 immune cell proportions in different ICDRGs clusters using the R package of “GSVA”.

### Establishment of gene-cluster subtypes

We utilized the ICDRG subgroup-based differential expressed genes (DEGs) to construct a gene cluster to investigate the functions of ICDRGs in GC. Firstly, we filtered the DEGs in the ICDRG clusters A, B, and C, using the “limma” package, with cutoffs of |fold change| > 1 and p < 0.05. Subsequently, we included the DEGs that intersected between the three clusters in the subsequent analysis. To analyze the potential functions of the ICDRG subgroup-based differential expressed genes, we further conducted the Kyoto Encyclopedia of Genes and Genomes (KEGG) and Gene Ontology (GO) analyses using the “ClusterProfiler” package. Additionally, we utilized the R package of “ConsensusClusterPlus” to separate the GC samples of ICDRG subgroup-based DEGs into two gene clusters.

### ICDRG score model establishment and validation

LASSO Cox regression analysis was utilized to identify ICDRGs with prognostic value, followed by multivariate Cox analysis to filter features and establish a ICDRG score model. The risk score for STAD patients was calculated using the formula: risk score =∑ (coefficients × expression of signature genes). Patients were divided into two groups named high- and low-risk groups according to the median of ICDRG score. A 7:3 division ratio was applied to split the GC samples into both training and test cohorts. To evaluate the accuracy of the score model in predicting the OS of GC patients, ROC curves were also analyzed.

### Prognostic analysis of ICDRG score model

Univariate and multivariate Cox analyses were further employed to identify potential prognostic factors for OS in GC. The ICDRG score signature and major clinical risk variables were evaluated using ROC curves to predict OS in GC patients. A nomogram was constructed by using the R packages named “rms” and “survival” to evaluate the clinical survival probability for STAD patients at 1-, 3-, and 5-year intervals, incorporating the ICDRG score and independent prognostic clinical parameters. Decision curve analysis (DCA) curves were performed using the “ggDCA” R package to assess the diagnostic accuracy of the nomogram, ICDRG score, and prognostic clinical parameters.

### Prediction of immunotherapy response and chemotherapeutic drugs

Tumor Immune Dysfunction and Exclusion (TIDE) was used to predict the immunotherapy responses for STAD patients by risk stratification (http://tide.dfci.harvard.edu). An Imvigor 210 (http://research-pub.gene.com/IMvigor210CoreBiologies) database was utilized to evaluate the PD-L1 immunotherapy response of GC samples. Based on the Genomics of Drug Sensitivity in Cancer (GDSC) (https://www.cancerrxgene.org/), the R package of “pRRophetic” was used to calculate the IC50 values of therapeutic drugs by the ridge regression.

### Cell culture

The human gastric cancer cell lines SGC-823 and SGC-7901 were both purchased from the Cell Bank of the Chinese Academy of Sciences (Shanghai, China). The cell culture conditions were maintained as follows: RPMI-1640 complete medium supplemented with 10% fetal bovine serum (FBS) and 1% antibiotics (100 U/mL penicillin and 100 ng/mL streptomycin) at 37° C in a humidified atmosphere of 5% CO_2_.

### Transient transfection

The SGC-823 and SGC-7901 cells were seeded in 6-well plates. Once the cell density reached 40% to 60%, transfections were made following the Lipofectamine 2000 transfection reagent instructions. The transfected cells were collected for further experimentation after 48 to 72 hours.

### CCK8 assay for cell viability

After terminating the digestion of the culture medium, the cells were centrifuged for 5 minutes. After discarding the supernatant, 3 mL of fresh medium was subsequently added to re-suspend the cells. The cells were then digested with trypsin, and after centrifugation and removal of the supernatant, the cells were resuspended. The cells were seeded in 96-well plates, with 5 × 103cells per well. After 24 hours of adherence, the cells were treated with interference of GPX1 and cultured until the designated time points (0, 24, 48, 72, 96 hours). According to the instructions, 10μl per 100ul serum-free culture medium of CCK8 solution (CCK-8; Biosharp, Shanghai, China) were added into the cells, and then incubated for 1 h at 37° C. Following that, absorbance was measured at 450nm with a microplate reader (BD Biosciences, USA). A growth curve was plotted based on the absorbance values and time.

### Clone formation experiments

SGC-7901 and SGC-823 cells were seeded in small dishes at a cell density of 1×10^3^ cells/mL, shaken, and placed in the incubator. After 5-7 days, under a microscope, the dishes were observed until each cell clone contained about 10-15 cells. The dishes were then removed, washed with PBS, fixed with methanol for 30 minutes, subsequently stained with crystal violet for another 10 minutes, and counted by taking photographs with a camera.

### Transwell experiment

SGC-823 and SGC-7901 cells were seeded in a serum-free culture medium (1×104 cells/100uL medium) into the upper chamber (8um pore size, Corning, USA). A culture medium containing 10% FBS (600 μL) was slowly added to the transwell plate lower chamber. After 24 hours of incubation, the invasive gastric cancer cells were fixed with 4% polyoxymethylene for 30 minutes at room temperature, subsequently followed by staining with 0.5% crystal violet for 10 minutes. The number of cells was counted in 5 randomly selected fields of view using a microscope.

### Cell scratch test

SGC-823 and SGC-7901 cells were seeded in a 6-well plate. Once the cells had completely covered the bottom of the wells, a scratch was made using a sterile pipette tip at the bottom of each well. The healing of the scratch was photographed under a microscope at 0 and 24 hours after the scratch was made. Three measurements of the scratch width were taken along the edge of the scratch, and the average value was calculated. The scratch healing rate (%) calculation was performed as follows: (initial scratch width - observed scratch width at the designated time point) / initial scratch width × 100%.

### Statistical analysis

R software (version 4.2.0) was used throughout this study to accomplish all statistical analyses. The Wilcoxon test was utilized for the two groups comparison, and one-way ANOVA testing was used to analyze differences for the group number that is over two. Analyzing the link between two variables was performed using the Spearman analysis. The significance levels were *P<0.05, **P<0.01, and ***P<0.001.

### Data availability statement

All data and clinical information involved in this paper were obtained from a public database (TCGA and GEO), approved by the Ethics Committee and written informed consent from patients was not required.

## RESULTS

### Potential role investigation of ICDRGs in GC

**Figure 1:**
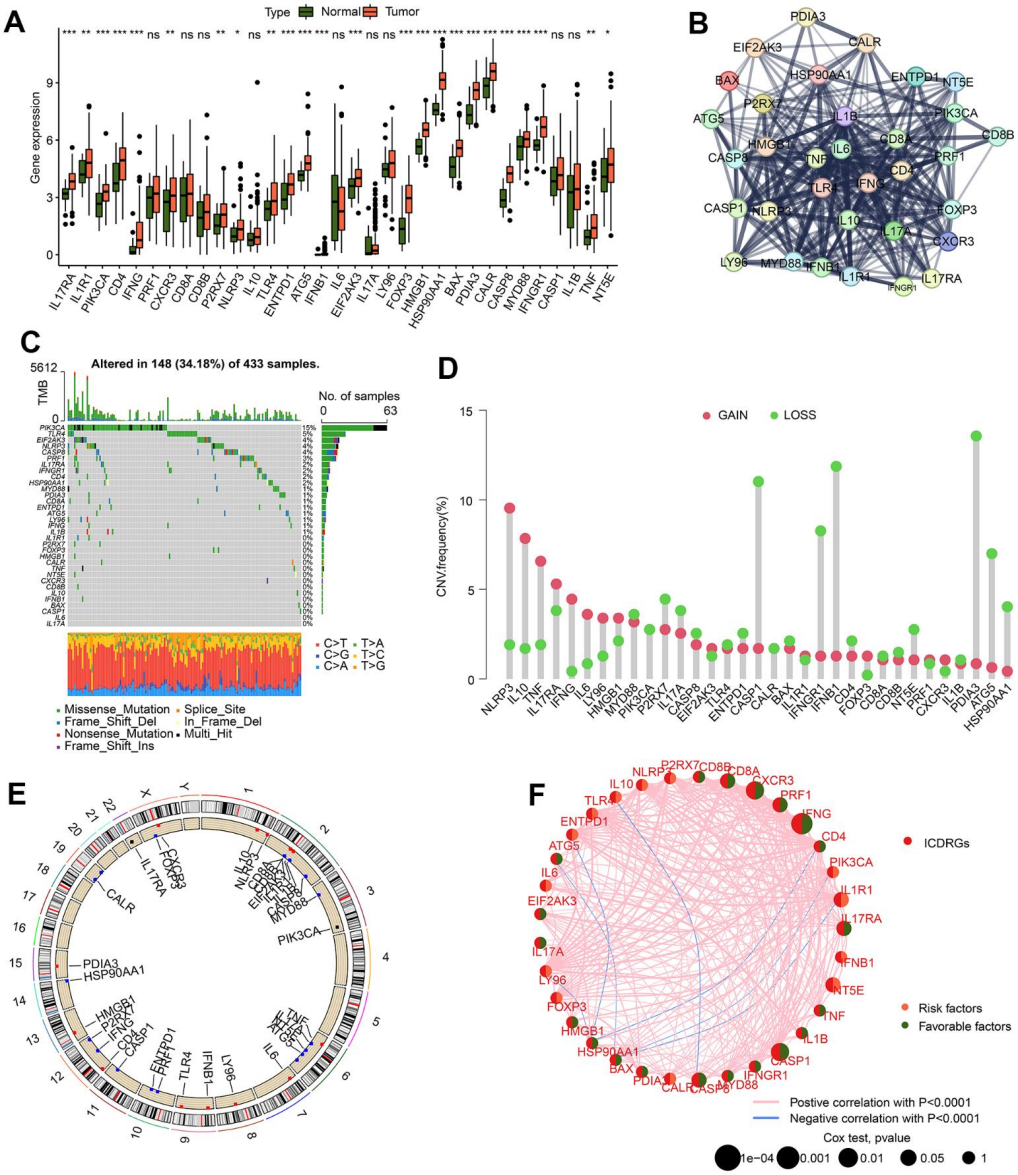
**The potential feature of ICDRGs in GC.** (**A**) Difference analysis of 33 ICDRGs in normal and GC tissues. (**B**) Interaction evaluation of 33 ICDRGs. (**C**) Mutation landscape of ICDRGs. (**D**) CNV estimation of ICDRGs in GC. (**E**) Circle diagram reveals the location of ICDRGs on chromosome. (**F**) Prognostic value and correlation analysis of ICDRGs.

We collected 33 ICDRGs to determine the potential function in the development of GC. After the estimation of difference analysis, a clear difference was observed in ICDRGs expression profile in normal and tumor tissues for GC, in which the GC samples had a greatly higher level of ICDRGs (Figure 1A). To learn more about the interaction of 33 ICDRGs, we used the STRING database to explore the potential association of ICDRGs. As implied in Figure 1B, an unambiguous relation was discovered between 33 ICDRGs predicted by the STRING database. The somatic landscape of ICDRGs displayed a distinct mutation frequency in 148 samples of 433 GC samples, in which the mutation frequency of PIK3CA, TLR4, EIF2AK3, NLRP3, and CASP8 was 15%, 5%, 4%, 4%, and 4%, respectively (Figure 1C). Moreover, the analysis of CNV revealed that the NLRP3, IL10, TNF, IFNG, IL6 and LY96 exhibited a higher amplification, whereas the CASP1, IFNGR1, IFNB1, PDIA3, ATG5 and HSP90AA1 showed higher deletion (Figure 1D). The location of ICDRGs on the chromosome was further explored and visualized in Figure 1E. Based on the univariate Cox analysis, the prognostic value of ICDRGs was estimated, and we obtained 9 prognostic features: IFNG, CXCR3, CASP1, PRF1, IL17RA, and CD8A were considered as favorable factors, and NT5E and IL1R1 were evaluated as risk factors (Figure 1F).

### ICDRG-based molecular subtypes development and immune infiltration assessment

**Figure 2:**
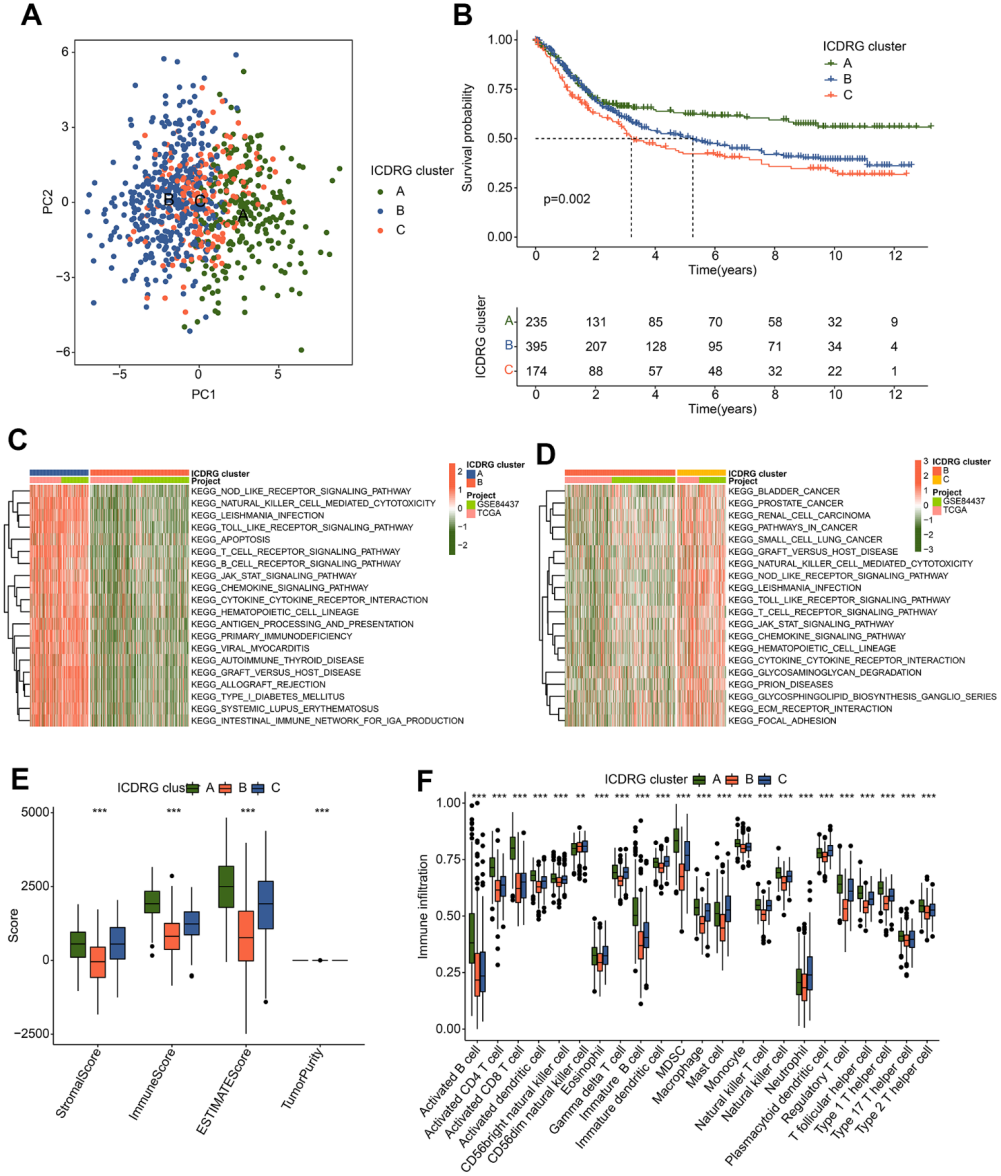
**Development of ICDRG-based molecular subtypes for GC.** (**A**) The PCA pattern of ICDRG-based molecular subtypes. (**B**) Clinical prognosis outcome of GC samples in ICDRG-based subgroups. (**C**, **D**) GSVA plot shows the dramatically altered KEGG signaling pathways between ICDRG-based subgroups. (**E**) ESTIMATE assessment of GC samples in ICDRG cluster A, B and C. (**F**) Immune infiltration investigation via ssGSEA algorithm.

To further determine the molecular subtypes of ICDRG for GC, we enrolled 804 GC samples from the TCGA-STAD and GSE84437 to develop an unsupervised consensus clustering analysis based on the expression profile of 33 ICDRGs. As illustrated in Figure 2A, we observed that the PCA plot could clearly differentiate the ICDRG cluster A, B, and C. The prognosis analysis of GC samples in ICDRG subtypes revealed that the clinical outcome of ICDRG cluster A was better than ICDRG cluster B and C (Figure 2B). To explore the underlying regulatory mechanisms responsible for the differences in clinical outcomes between the ICDRG subtypes, the GSVA was employed to estimate the difference in KEGG signaling pathways for GC. Between ICDRG cluster A and B, immune-related regulatory pathways were clearly down-regulated in the ICFRG cluster B for GC, involving in NOD-like receptor signaling pathways, natural killer cell-mediated cytotoxicity and toll-like receptor pathway signaling (Figure 2C). Of note, we discovered that a series of tumor association function was greatly enriched in the ICDRG cluster C of the GC sample, such as bladder cancer, prostate cancer, renal cell carcinoma, and pathways in cancer (Figure 2D). Considering the GSVA results in ICDRG subtypes, the immune infiltration was assessed of GC samples in the ICDRG subgroups. The ESTIMATE analysis suggested a conspicuous difference in ESTIMATE evaluation in ICDRG cluster subgroups (Figure 2E). On the basis of the ssGSEA assessment algorithm, we detected that the fraction of most 23 immune cells was greatly higher of GC samples in ICDRG cluster A, such as activated B cell, MDSC, immature B cell, and CD8 + T cell (Figure 2F). These discoveries demonstrate that the ICDRG expression characteristic could accurately classify the GC samples into different molecular subtypes and closely related to immune infiltration.

### Establishment of gene-cluster subtypes based on the ICDRG subgroup-based DEGs

**Figure 3:**
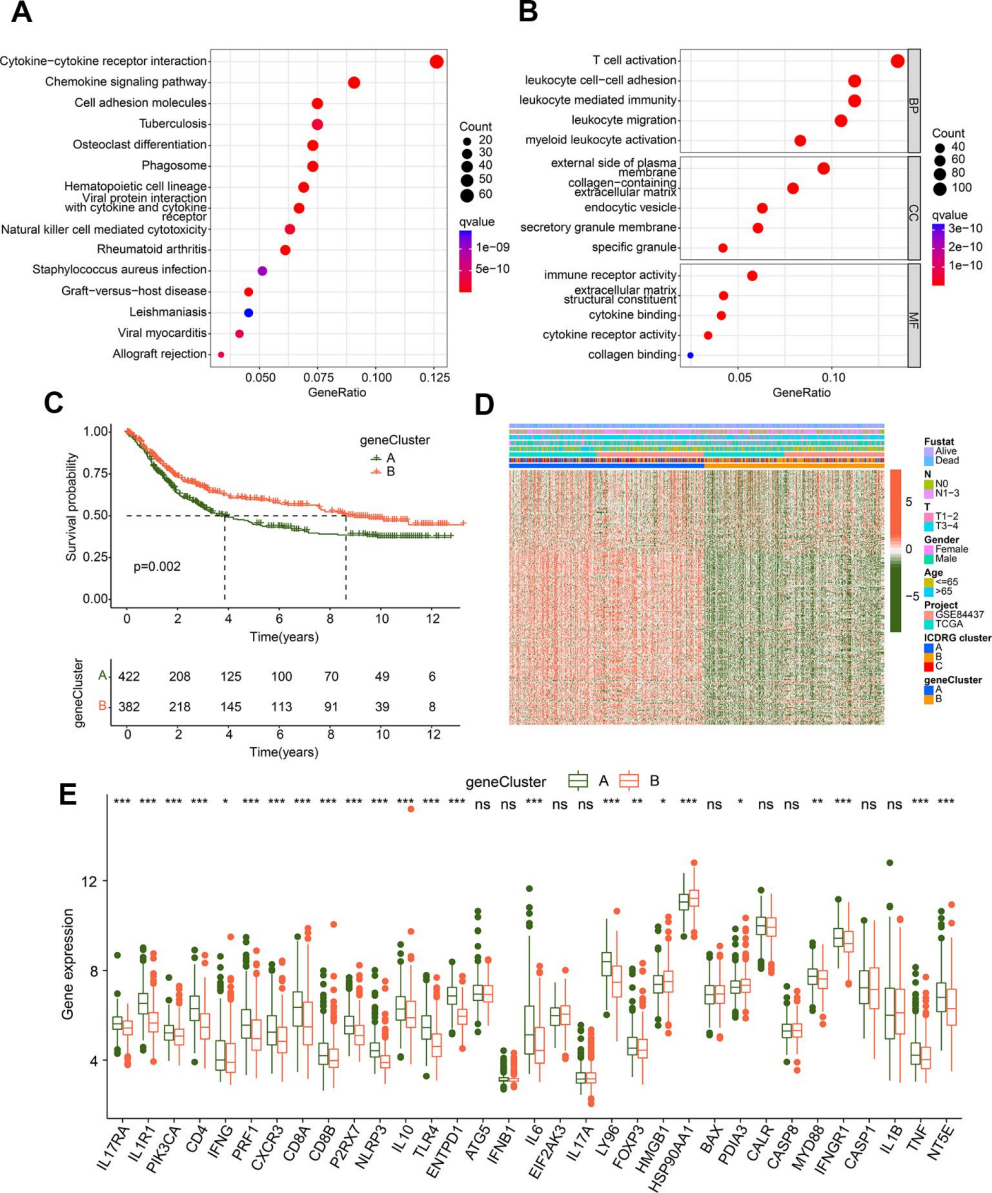
**Generation of gene-cluster subgroups based on the ICDRG subtypes-based DEGs.** (**A**, **B**) KEGG and GO analysis of ICDRG subtypes-based DEGs. (**C**) Prognosis analysis of GC samples in gene-cluster subgroups. (**D**) Heatmap shows the relationship between prognostic DEGs expression and clinical variates. (**E**) Expression profile of 33 ICDRGs in the gene-cluster subgroups.

To further explore the biological mechanism of ICDRG subgroups, we explored the DEGs between ICDRG cluster A, B and C. With the criterion cutoff set as |fold change| > 1 and p < 0.05, 876 overlapping DEGs between ICDRG subgroups were obtained (Supplementary Figure 1). The KEGG analysis of DEGs implied that cytokine-cytokine receptor interaction, chemokine signaling pathway, and cell adhesion molecules were observably enriched (Figure 3A). GO bubble diagram illustrated that T cell activation, leukocyte cell–cell adhesion, immune receptor activity, and external side of plasma membrane were enriched with DEGs (Figure 3B). Those enrichment results suggested that immune-associated function may participate in the role of ICDRG subgroup-based DEGs in the progression of GC. Thereafter, those DEGs were enrolled to explore the prognosis implications for GC samples via univariate Cox analysis, and 244 prognostic variates were collected in total. On the basis of 244 prognostic variates, an unsupervised consensus clustering analysis was carried out to classify the GC samples into 2 gene-cluster subgroups, with 422 samples in gene-cluster A and 382 samples in gene-cluster B. The analysis of clinical outcome revealed that the survival rate of GC samples in gene-cluster A was worse than in gene-cluster B (*p* = 0.002, Figure 3C). The heatmap plot implied the relationship between 244 prognostic variates expression profile and clinical features, and the result showed the expression level of 244 prognostic variates was greatly lower in gene-cluster B for GC (Figure 3D). The expression of ICDRGs suggested that most of ICDRGs were upregulated of GC samples which with poor prognosis, such as IL17RA, IL1R1, PIK3CA, and CD4 (Figure 3E).

### Generation of ICDRG score based on the ICDRG subtypes-based prognostic DEGs

**Figure 4:**
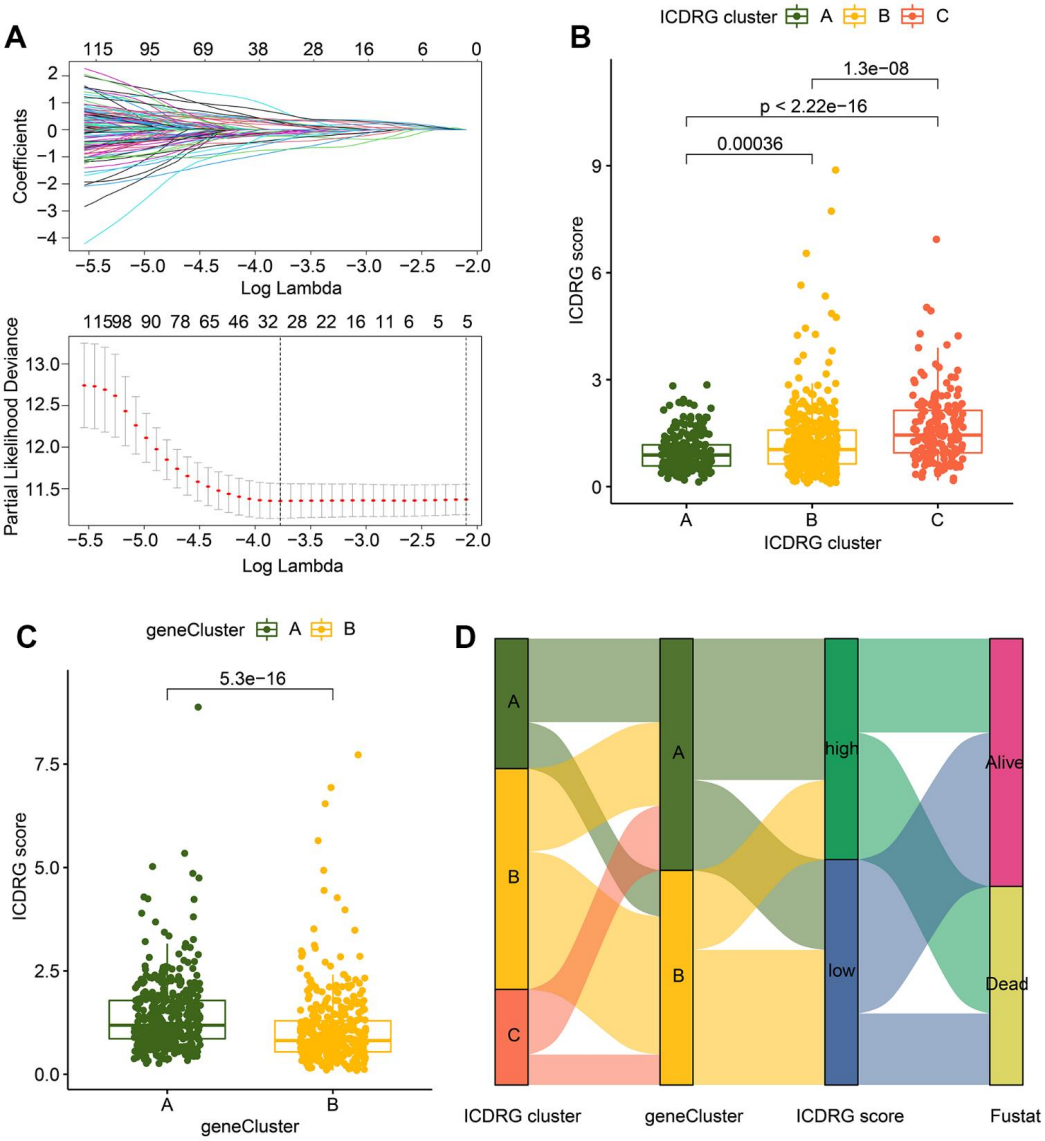
**Establishment of ICDRG score for GC samples.** (**A**) LASSO analysis for selecting characteristic variates to construct ICDRG score model. (**B**) The distribution of ICDRG score in the ICDRG subgroups. (**C**) Analysis of ICDRG score in gene-cluster subgroups. (**D**) The Sankey diagram reveals the association of ICDRG score, clinical status, ICDRG cluster subgroup and gene-cluster subtypes.

The ICDRG score was evaluated based on the ICDRG subtypes-based DEGs to divide the GC into different risk subgroups. The LASSO analysis selected 31 feature variables from the ICDRG subtypes-based prognostic DEGs for the subsequent analysis (Figure 4A). Multivariate Cox analysis identified 17 momentous variables to establish the ICDRG score model. In the ICDRG cluster subgroup, a noteworthy difference was observed between the ICDRG cluster subgroups, which the ICDRG score of GC samples in ICDRG cluster C was conspicuously higher than other ICDRG subgroups (Figure 4B). In the gene-cluster subgroups, we also found that the GC samples with poor clinical prognosis in the gene-cluster A had higher ICDRG score than gene-cluster B (Figure 4C). As displayed in Figure 4D, The Sankey plot revealed the relationship between ICDRG score, clinical status, ICDRG cluster subgroup, and gene-cluster subgroup. In summary, those discoveries demonstrate that the ICDRG score developed of cluster-related DEGs is closely associated with the prognosis for GC and could distinguish the GC samples into different risk subgroups in ICDRG molecular subtypes and gene-cluster subtypes.

### Development and verification of ICDRG model for GC

**Figure 5:**
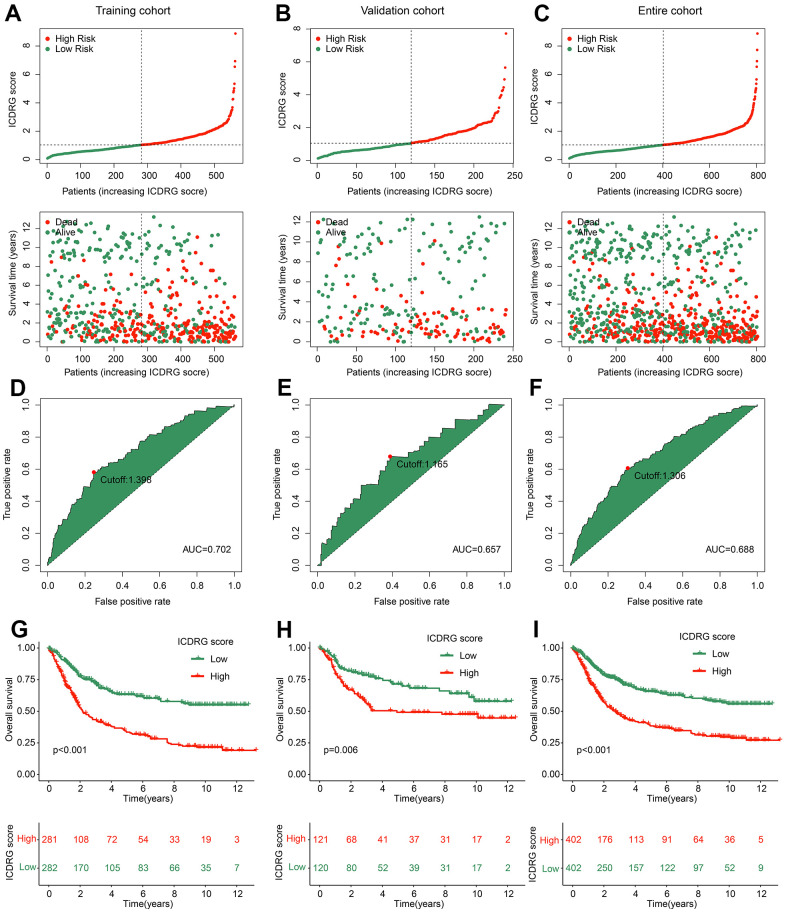
**ICDRG score model construction and clinical prognosis analysis.** (**A**–**C**) ICDRG score distribution in training, validation and entire cohorts. (**D**–**F**) ROC curve of ICDRG score. (**G**–**I**) Clinical prognostic outcome of GC samples in the training, validation and entire cohorts.

We developed a risk model to predict the clinical prognosis for each GC sample based on the ICDRG score. Under the division cutoff of 7:3, the GC samples were assigned into training and test cohorts, respectively. According to the median ICDRG score, the GC samples in the training, test, and entire cohorts were classified into low- and high ICDRG score subtypes (Figure 5A–5C). The results implied that the GC samples with high ICDRG scores observably tend to lower survival times. ROC analysis of ICDRG score in the training, test and entire cohorts was 0.702, 0.657, and 0.688, respectively (Figure 5D–5F). Clinical prognosis curve analysis implied that the clinical outcome of GC samples with high ICDRG scores was observably lower than those with low ICDRG scores in the training, test, and entire cohorts (Figure 5G–5I). We thus speculate that ICDRG scores could accurately distinguish the GC samples into two risk subtypes, and the high ICDRG score is associated with poor prognosis in GC.

### Comprehensive analysis of independent prognosis for ICDRG score in GC

**Figure 6:**
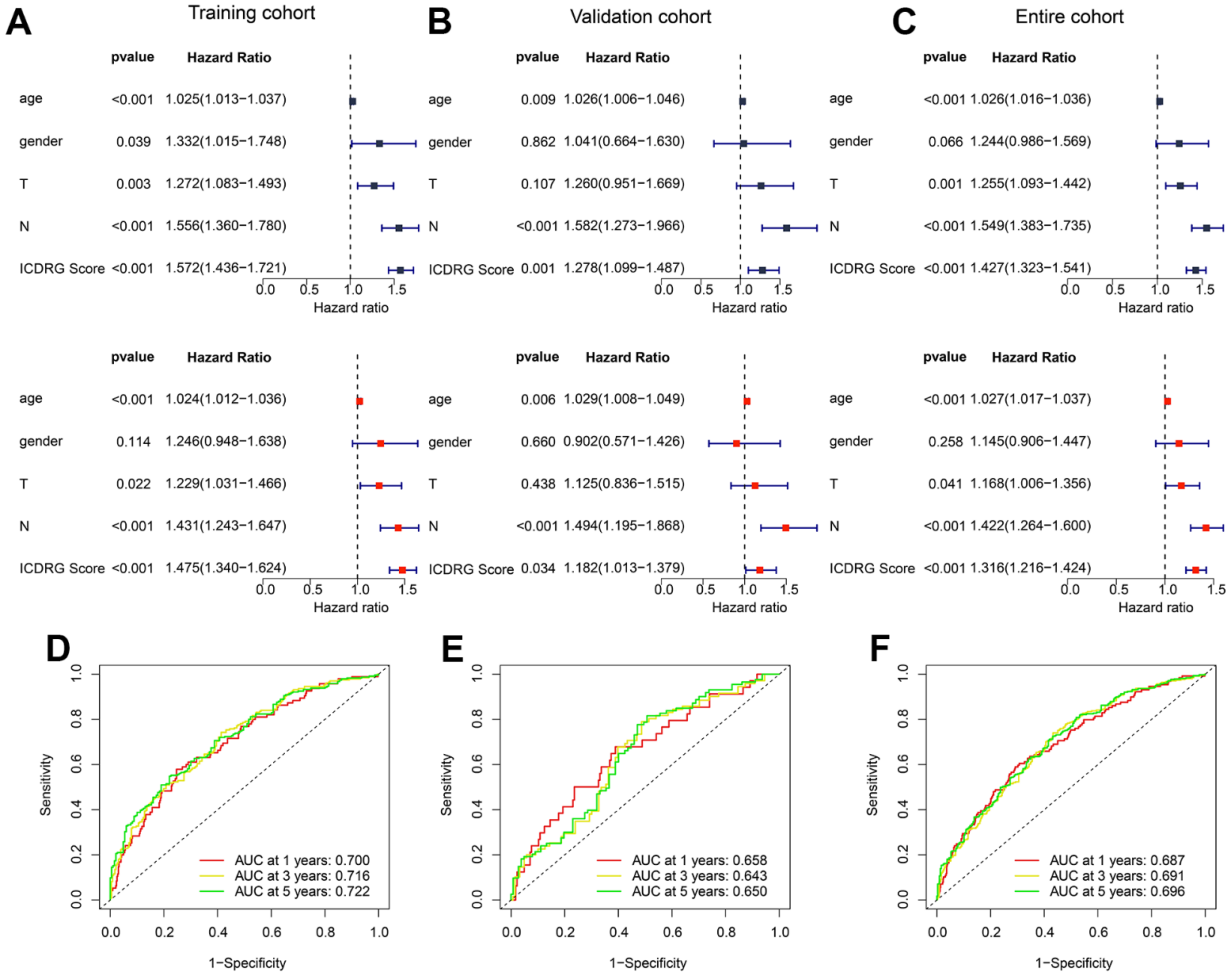
**Independent prognostic analysis of ICDRG score and clinicopathological features in GC.** (**A**–**C**) The univariate and multivariate Cox analysis of ICDRG score and clinical features for GC in the training, test and entire cohorts. (**D**–**F**) ROC analysis of 1-, 3-, and 5-years in the training, test and entire cohorts.

In the view of ICDRG in predicting clinical prognosis for GC, we comprehensively analyzed the independence of ICDRG and different clinicopathological features in GC. In the training cohort, the results of univariate Cox analysis (p < 0.001, HR = 1.572(1.438-1.721)) and multivariate Cox analysis (p < 0.001, HR = 1.475(1.340-1.624)) displayed that the ICDRG score was explored as a risk factor with poor clinical prognosis (Figure 6A). In the test cohort, we observed that the ICDRG score was considered a poor prognosis factor via the univariate Cox analysis (p = 0.001, HR = 1.278(1.099-1.487)) and multivariate Cox analysis (p = 0.034, HR = 1.182(1.013-1.379)) (Figure 6B). In the entire cohort, the results of univariate Cox analysis (p < 0.001, HR = 1.427(1.323-1.541)) and multivariate Cox analysis (p < 0.001, HR = 1.316(1.218-1.424)) also demonstrated that the ICDRG score was an independent prognosis predictor which related to the poor clinical outcome for GC (Figure 6C). ROC curve analysis revealed that the AUC of 1-, 3-, and 5 years was 0.700, 0.716, and 0.722 in the training cohort, 0.658, 0.643 and 0.650 in the test cohort, 0.687, 0.691 and 0.696 in the entire cohorts, respectively (Figure 6D–6F).

### Nomogram establishment of ICDRG score and clinical features for GC

**Figure 7:**
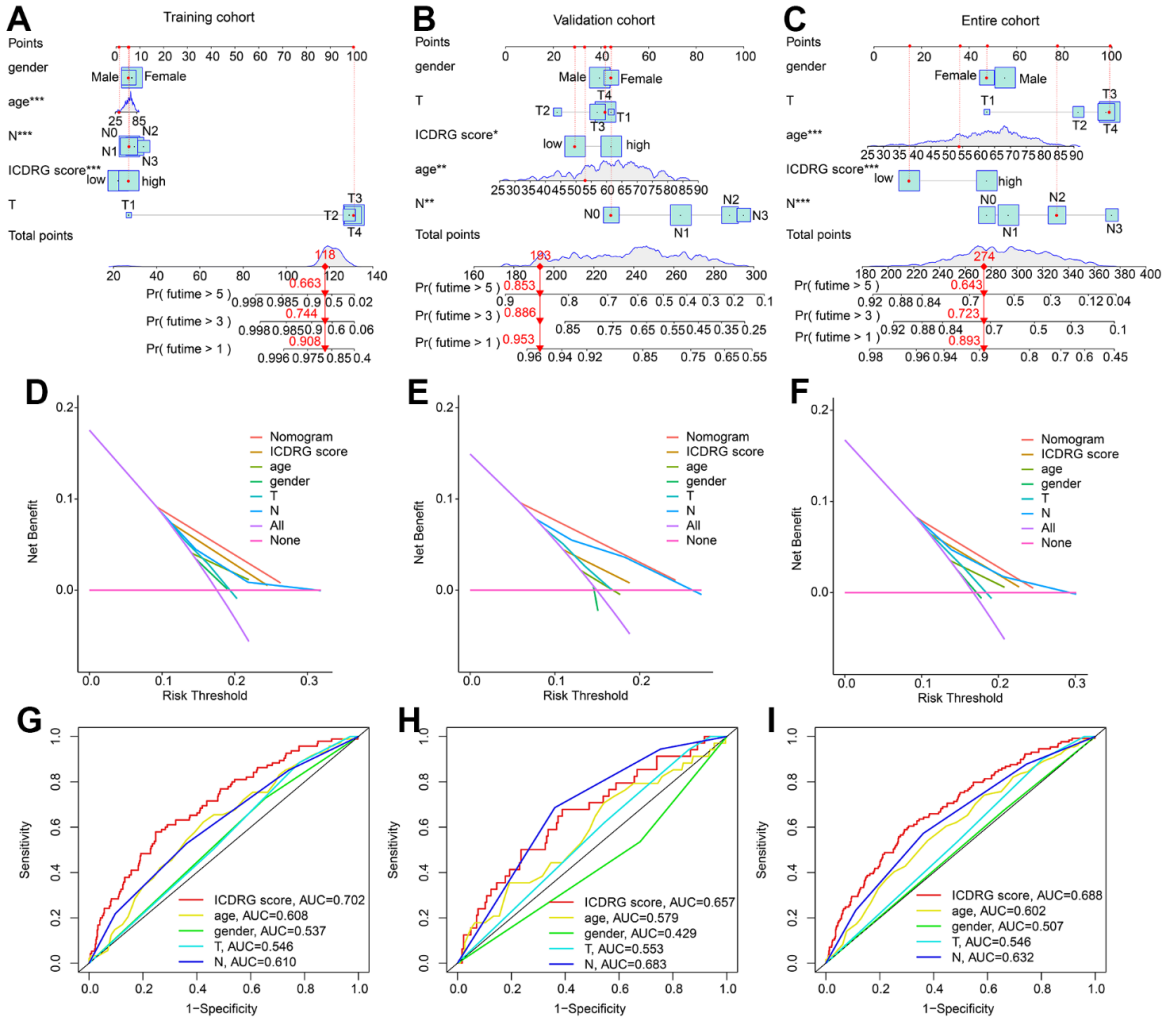
**Development of nomogram based on the ICDRG score and clinical features in GC.** (**A**–**C**) Nomogram construction based on the ICDRG score and GC-related clinical parameters in the training, test and entire cohorts. (**D**–**F**) DCA model shows the accuracy of ICDRG score and other GC-related clinical parameters in evaluating clinical prognosis for GC. (**G**–**I**) Diagnostic power analysis of ICDRG score and GC-related clinical parameters.

Based on of ICDRG score and clinical features, we established a nomogram to estimate the clinical survival outcome of GC samples in 1-, 3-, and 5 years. As displayed in Figure 7A–7C, the nomogram analysis illustrated that the ICDRG score could accurately evaluate the clinical outcome of GC samples in the training, test and entire cohorts. The results of the DCA curve displayed that the accuracy of the nomogram in predicting clinical prognosis for GC in the training, test, and entire cohorts were noteworthily better than other parameters (Figure 7D–7F). Compared to the other clinical parameters, the AUC of ICDRG score in the training and entire cohorts was higher, indicating a favorable diagnostic power than the clinical features (Figure 7G–7I).

### Immune infiltration analysis in ICDRG score subtypes

**Figure 8:**
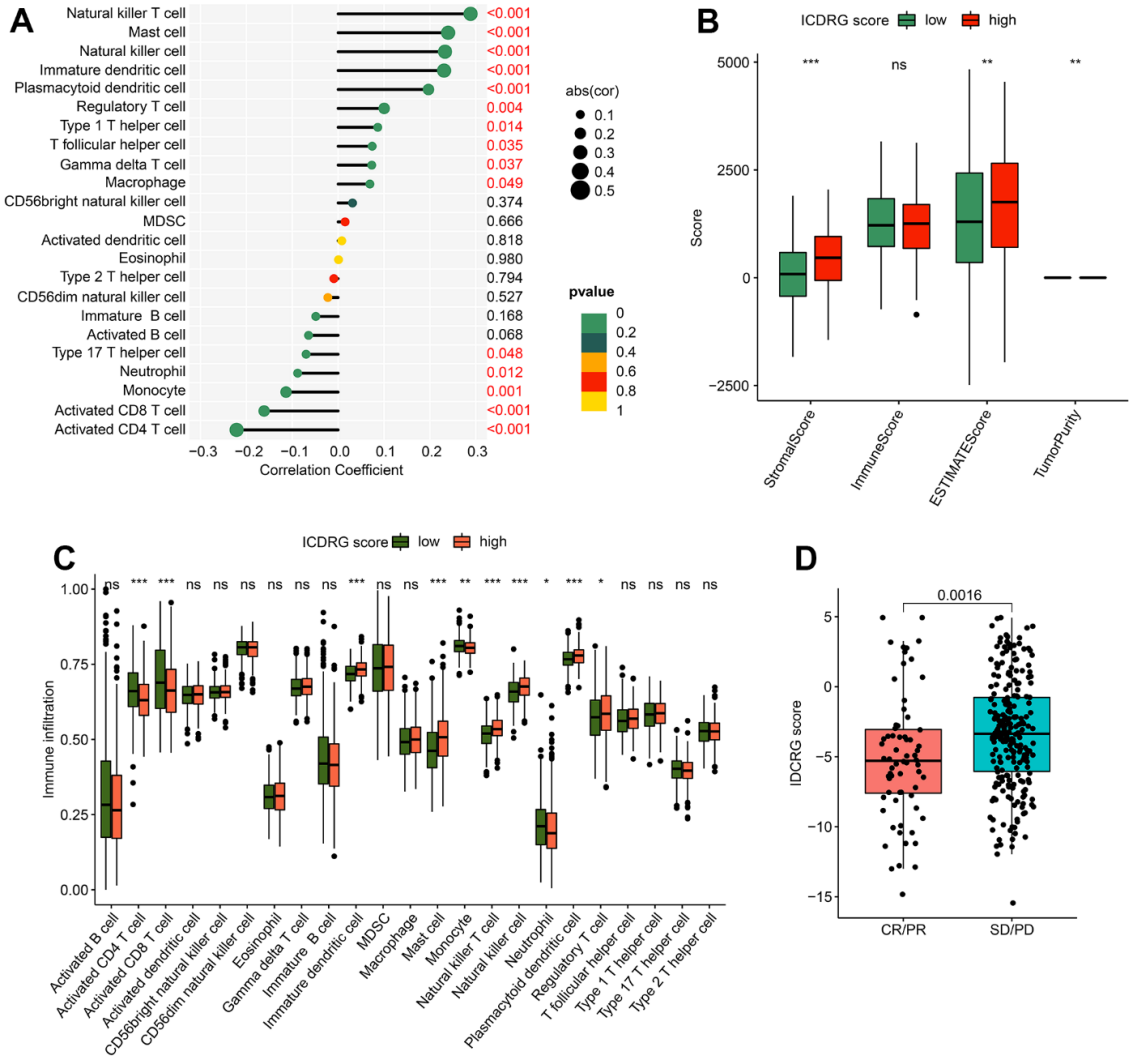
**Immune infiltration analysis and immunotherapy response in ICDRG score subtypes.** (**A**) Correlations between the ICDRG score and immune cell infiltration. (**B**) ESTIMATE assessment. (**C**) Distribution of 23 immune cells in the high- and low- score groups. (**D**) Relationship of the ICDRG score and anti-PD-L1 immunotherapy response.

The potential relationship of ICDRG score and immune infiltration was further explored and the result implied that the ICDRG score was positively correlated with macrophage, gamma delta T cell, T follicular helper cell, type 1 T helper cell, regulatory T cell, plasmacytoid dendritic cell, immature dendritic cell, natural killer cell, mast cell, and natural killer T cell. However, a noteworthy negative correlation was observed between ICDRG score and CD4+ T cell, CD8+ T cell, monocyte, neutrophil, and type 17 T helper cell (Figure 8A). According to the ESTIMATE assessment algorithm, the stromal and ESTIMATE score were higher in the high ICDRG score group, but the tumor purity was lower in the high ICDRG score group (Figure 8B). Using ssGSEA, we investigate the different distribution of 23 immune cells in the high- and low-risk groups. The results revealed that the high-risk group had higher proportions of macrophages, natural killer cells, and plasmacytoid dendritic cells while having lower proportions of activated CD4 T cells, activated CD8 T cells, and neutrophils. (Figure 8C). In addition, we found that the ICDRG score had significantly higher stable/progressive disease (SD/PD) compared to responders (CR/PR) (Figure 8D).

### Chemotherapy drug prediction in ICDRG score subgroups

**Figure 9:**
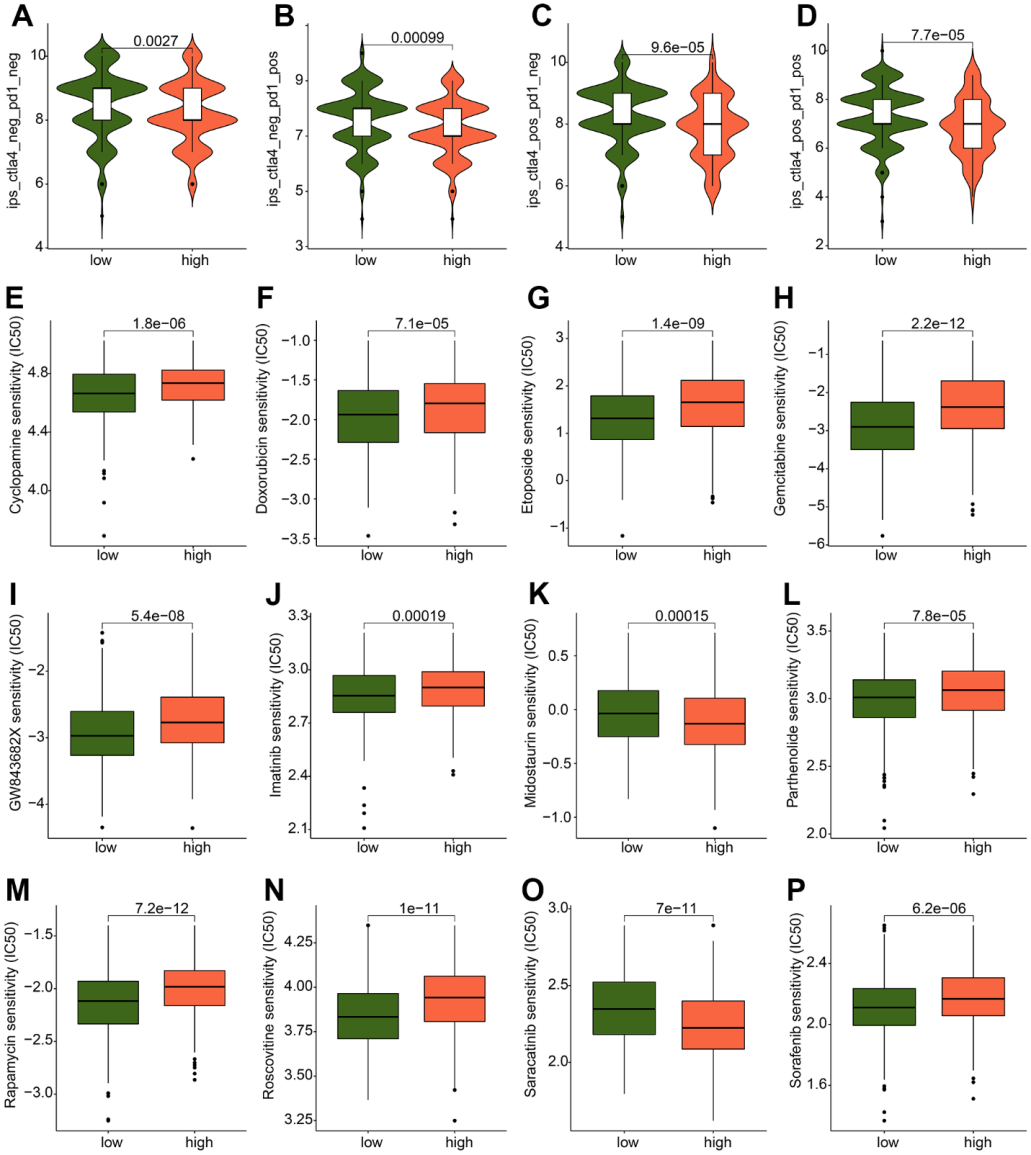
**Drug sensitivity analysis between the ICDRG score subtypes.** (**A**–**D**) IPS evaluation shows the response to PD-1 and CTLA-4 of GC in ICDRG score subtypes. (**E**–**P**) Prediction of chemotherapy drug for GC in ICDRG score subgroups.

The IPS results revealed that the GC patients in the low-risk group were more sensitive to the PD-1, CTLA-4, and PD 1/CTLA-4 treatment (Figure 9A–9D). Moreover, we discovered that the ICDRG score subgroups respond differently to chemotherapeutic medicines based on the IC50 calculation. In detail, the high-risk score had lower IC50 levels for midostaurin and saracatinib, indicating that chemotherapy may have a greater impact on high-risk patients. While, the GC patients with low-risk score were more sensitive to cyclopamine, doxorubicin, etoposide, gemcitabine, GW843682X, imatinib, parthenolide, rapamycin, roscovitine and sorafenib (Figure 9E–9P). These findings suggest that the ICDRG score might be immune-related and might be able to predict the GC chemotherapy therapy.

### Connection between ICDRG score and landscape of somatic mutations

**Figure 10:**
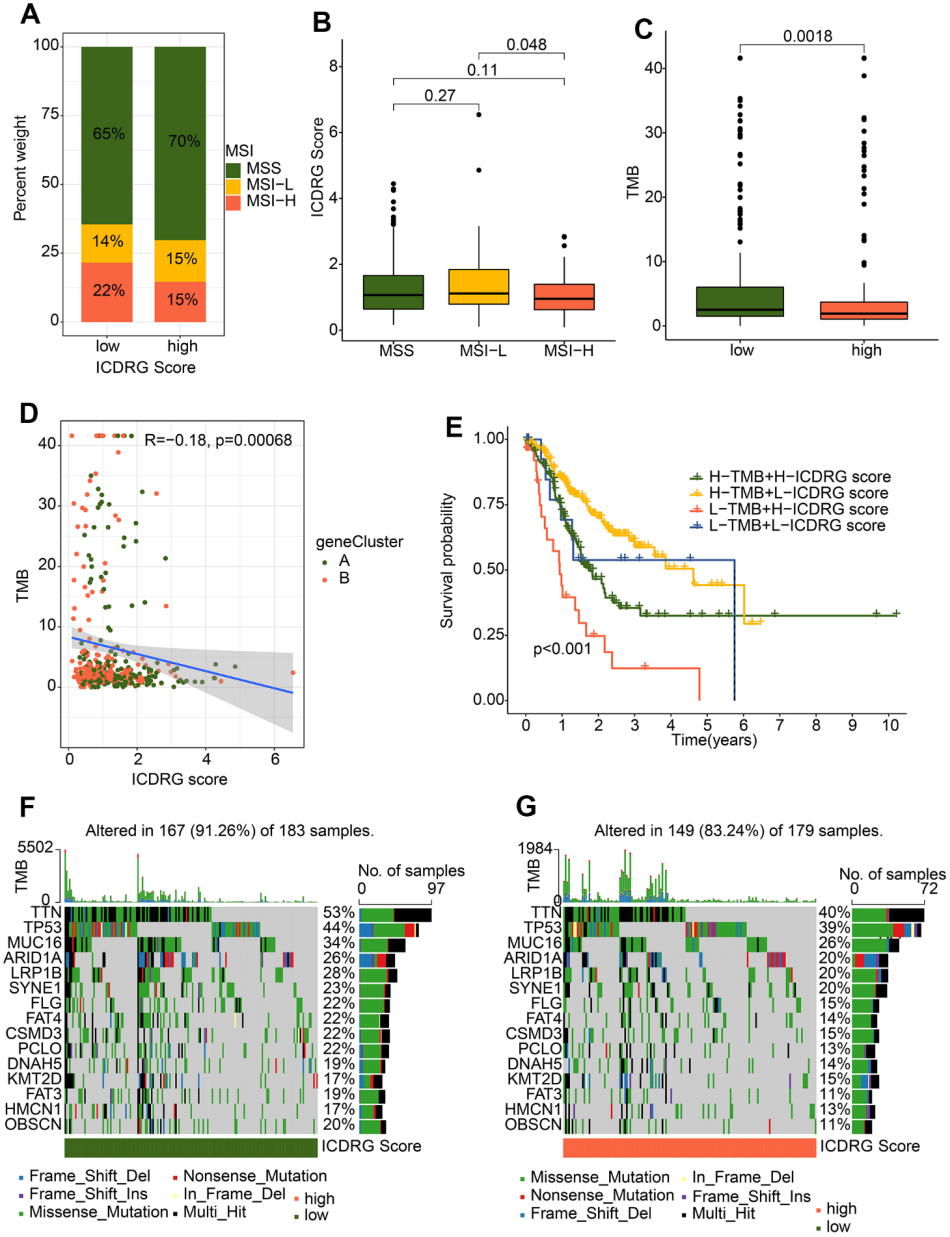
**Somatic mutation landscape and MSI in GC.** (**A**) Percent of MSI in low- and high-risk groups. (**B**) Distribution of risk score in MSS, MSI-L, and MSI-H. (**C**) TMB analysis. (**D**) Correlation analysis of TMB and ICDRG score. (**E**) Kaplan–Meier plotter for GC patients stratified ICDRG score and TMB. (**F**, **G**) The top 15 most frequently mutated genes in low- and high-risk groups.

In this study, we analyze the interaction between the ICDRG score and the landscape of somatic mutations. At first, we investigated the percentage of MSI in the high and low ICDRG score subgroups, and we discovered that the high-score group had a larger percentage of MSI-L and a lower percentage of MSI-H. (Figure 10A). Importantly, GC patients with MSI-H had risk scores much lower than those with MSS and MSI-L (Figure 10B). To further investigate, we examined tumor mutation burden (TMB) values between the high and low ICDRG score subgroups and discovered that TMB was significantly lower in the ICDRG score group (Figure 10C). Subsequent correlation analysis displayed that the TMB was negatively linked with the ICDRG score (Figure 10D). Therefore, we combined TMB and ICDRG score served as a prognostic indicator, the differences in overall survival between different groups were obvious. As demonstrated by Kaplan-Meier analysis, we validated the cooperative effect of two indicators in the prognostic prediction of GC and there was no interference of the TMB status with the ICDRG scores in the prognostic predictive performance. ICDRG scores subgroups in high-score group exhibited worse prognosis in both low and high TMB status subtypes (p < 0.001) (Figure 10E). In addition, we showed comprehensive patterns of somatic variants and listed the top 15 most common mutated genes in the high- and low-risk groups. In the low-risk group, the top 5 mutated genes were TTN (53%), TP53 (44%), AUC16 (34%), ARID1A (26%), and LRP1B (28%), which exhibited high mutation frequencies than high-risk group (Figure 10F, 10G). Taken together, these results suggest that ICDRG score may act as an independent prognostic indicator and potential drug treatment target.

### Knockdown of GPX1 inhibits the proliferation, migration and invasion of gastric cancer cells

**Figure 11:**
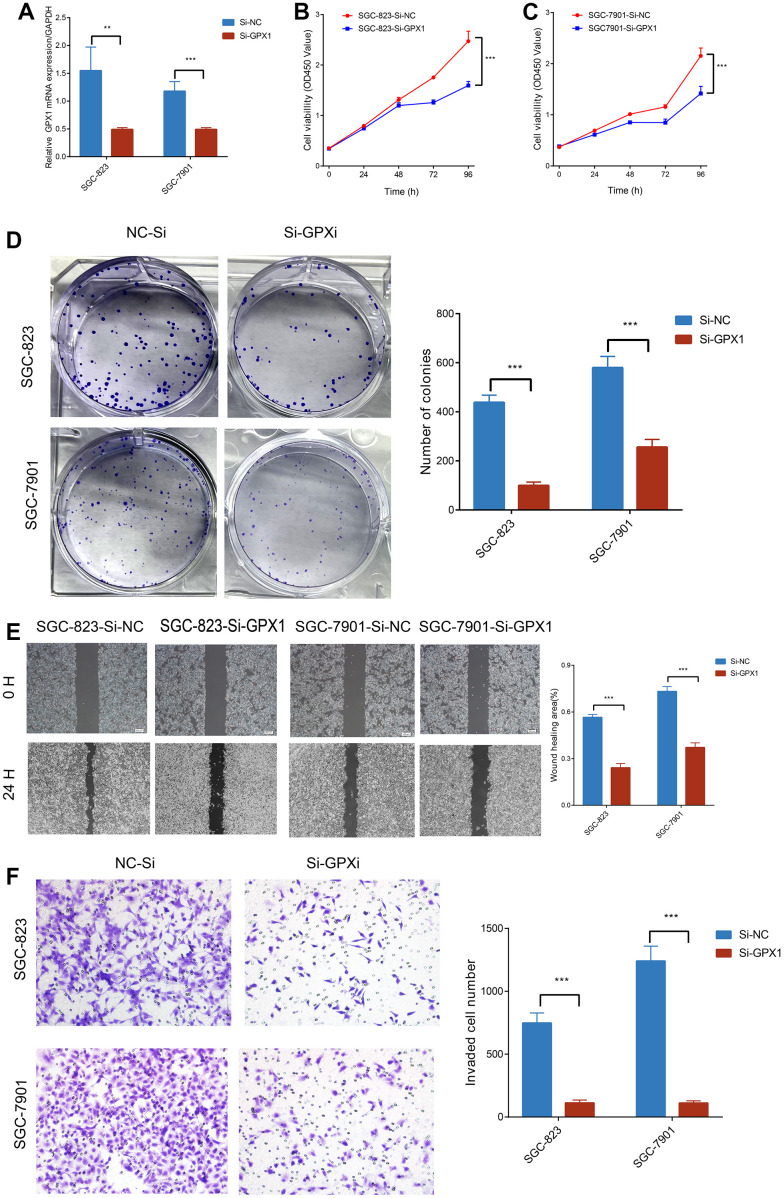
**The impact of GPX1 on the migration, invasion, and proliferation of gastric cancer cells.** (**A**) Relative expression of GPX1 detected by qPCR in SGC-823 and SGC-7901 cells (n=3). Cell viability of (**B**) SGC-823 and (**C**) SGC-7901 cells after being treated with Si-NC and Si-GPX1 (n=3). (**D**) Clone formation experiments (n=3). (**E**) Scratch assays. (n=3) (×40). (**F**) The numbers of SGC-823 and SGC-7901 cells that traversed the transwell membrane (n=3) (×200).

To further investigate the role of GPX1 in gastric cancer development, we transfected GPX1 siRNA into human gastric cancer cell lines SGC-823 and SGC-7901. qRT-PCR results showed that GPX1 was significantly downregulated compared to the negative control group (Figure 11A). Firstly, we detected cell proliferation activity using CCK8 at 24, 48, 72, and 96 hours, and found that the proliferation ability of SGC-823 and SGC-7901 cells was significantly lower than that of the control group at 72 and 96 hours (Figure 11B, 11C). We also observed that knocking down GPX1 significantly inhibited the colony formation ability of SGC-823 and SGC-7901 cells (Figure 11D). Next, we conducted scratch and transwell assays to evaluate the role of GPX1 in gastric cancer migration. Compared with the control group, the scratch continued to heal over time, and knocking out GPX1 inhibited the ability of cells to migrate into the scratch area (Figure 11E). Similarly, transwell analysis showed that knocking down GPX1 significantly reduced the number of SGC-823 and SGC-7901 cells that penetrated the lower chamber (Figure 11F). These results indicate that knocking down GPX1 can inhibit the proliferation, migration, and invasion ability of gastric cancer cells.

## DISCUSSION

In this study, we established a risk model for STAD patients and explored the possible mechanisms of the difference in prognosis of STAD by means including immune infiltration, mutation burden analysis, drug resistance analysis.

Our results show that the prognostic model established based on ICDRGs can effectively predict the prognosis of patients with STAD. ICDRGs have been shown to participate in tumor progression and are associated with anti-tumor therapy responsiveness in a variety of tumor types. Immunogenic death inducers can induce CD8^+^ T cell-dependent anti-tumor immunity to enhance tumor immunotherapy [21]. In GC, adjuvant chemotherapy regiments containing the ICD inducer oxaliplatin altered immune cell invasion and subtype by significantly reducing FOXP3^+^ Treg cells and increasing the diversity of CD8^+^ cytotoxic T cells and TCR in GC [22]. In addition, radiotherapy combined with 5-FU upregulated immunogenic cell death related molecules and increased the expression level of PD-L1 [23]. Therefore, the combined therapeutic strategy of ICD inducers and immune checkpoint inhibitors theoretically contributes to a better GC prognosis.

We have verified the effect of GPX1 on the proliferation, migration and invasiveness of GC cells through *in vitro* experiments, thus partially proving the reliability of our bioinformatics results. In previous reports, 16% of GC patients showed abnormal methylation of GPX1 [24]. GPX1’s correlation with the risk of GC attacks also suggests a role in the development of GC [25]. Further mechanism studies revealed that the interaction of Ga-binding protein α (GABPA) with GPX1 may be one of the causes of GC progression [26]. According to the current evidences, GPX1 acts as a tumor promoter in GC patients. However, GPX1 has a complex dichotomous role as a potential tumor suppressor or promoter in different cancers, given that it is involved in various signaling pathways to regulate multiple tumor-related biological behaviors [27]. Therefore, an in-depth and comprehensive mechanism study is still needed to determine the role of GPX1 in GC.

Disruption of the balance between immunosuppression and immune activation signals has a significant impact on the progression of GC and patient outcomes [28]. Long-term chronic inflammatory manifestations in the presence of GC microenvironments promote tumor progression and reduce treatment opportunities [29]. Multicomponent immune cells, including lymphocytes, NK cells and macrophages show their involvement in the GC process [30]. In addition, a variety of immune-related signaling pathways, including HIPPO, Notch and Wnt, are involved in GC development [31–33]. Based on these known pathways and immune-mechanisms, a variety of immune-related therapies have been developed with clinical application potential for GC patients, including dendritic cell-based vaccines. adoptive T cell transfer cytokines and checkpoint inhibitors, etc. [34] Our results also confirmed the importance of immune-related metrics. By subgroup analysis, we found that the immune score and its related ESTIMATE score were higher in cluster A group, which had a relatively good prognosis. This immune score system based on the composition of immune cells in tumor tissue can improve the accuracy of GC survival prediction and is an important supplement to the AJCC staging system for stage II/III GC patients [35, 36]. In addition, a combination of immune score and TNM staging had a better prognostic value than TNM staging alone [37]. In 2021, immune score was further confirmed its impact on predicting the therapeutic effect of gastric cancer patients receiving adjuvant chemo/radio-therapy [38]. However, it remains unclear how immune score can be used to guide GC treatment. In colon cancer patients, the study revealed that high immune score level patients benefited from more cycles of adjuvant chemotherapy, while those with low immune score levels did not [39]. This may indicate the need for more aggressive treatment of GC patients with high immune score in order to achieve better outcomes. Since no similar results are published in GC patients, future large-scale analyses of GC, immune score and efficacy of treatment will help further evaluate the clinical value of immune score.

During the subgroup analysis, cluster A and cluster C with a good prognosis and a poor prognosis had a higher level of eosinophils, while cluster B had a lower level of eosinophils expression. The inconsistency of eosinophils expression level and prognosis caught our attention. Unforeseen effects of eosinophils have been found in a variety of biological processes beyond allergic inflammation, including carcinogenesis [40]. Eosinophils infiltrate a variety of tumors, and may regulate tumor progression directly by interacting with tumor cells or indirectly by forming TME. Depending on the type of tumor, eosinophils may have pro-tumor or antitumor functions [40]. Eosinophils have been reported to exert antitumor effects in gastric cancer [41]. Eosinophils produce several chemokines that are essential for the attractiveness of CD8 T cells in the TME [42]. In addition, eosinophils favor macrophage M1 production over M2 production through IFN-γ and TNF-α production during macrophage polarization [43]. The above evidence supports the antitumor effect of eosinophils in GC. However, studies on eosinophils in GC are far from in-depth. In fact, the functional plasticity of eosinophils depends on environmental factors that may vary in different microenvironments of cancer types, or even individual differences [44]. In addition, a literature reported that a high level of eosinophils infiltration in GC was a marker of optimal prognosis [45]. These paradoxical results call for further study of eosinophil function in GC.

The study also has several limitations. As a retrospective study based on a public database, it is difficult to cover differences across geographic areas. *In vitro* experiments without clinical validation makes the validation of public database inadequate. Additionally, the transcriptome profiles used in this study were all derived from core samples of tumor tissue. Given the fact that the microenvironment may be different in different tumor regions, it is impossible to recognize the differences between the core and the invasive marginal zone. A large, international, comprehensive, multicenter clinical study will help to further validate our findings in the future.

## Supplementary Material

Supplementary Figure 1

Supplementary Table 1
