# Supplementary material for: Development of an immunogenic cell death prognostic signature for predicting clinical outcome and immune infiltration characterization in stomach adenocarcinoma
Source: Aging (Albany NY). 2023 Oct 19;15(20):11389–411. doi: 10.18632/aging.205132 (PMC10637829; doi:10.18632/aging.205132)
Supplement: Supplementary Figure 1 [file aging-15-205132-s001.pdf]

SUPPLEMENTARY FIGURE

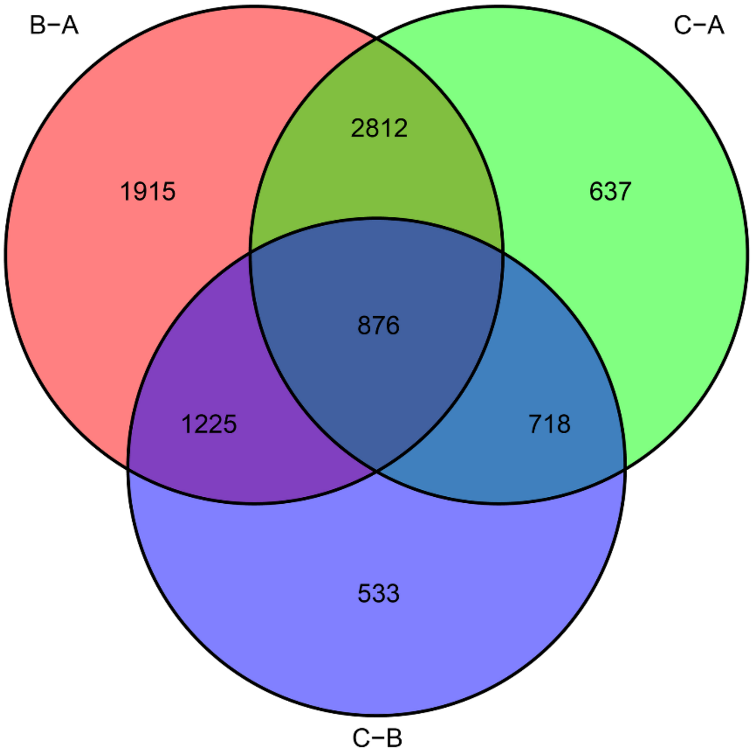

Supplementary Figure 1. Identification of the DEGs of ICDRG-based molecular subtypes.
