# Supplementary material for: Development of an immunogenic cell death prognostic signature for predicting clinical outcome and immune infiltration characterization in stomach adenocarcinoma
Source: Aging (Albany NY). 2023 Oct 19;15(20):11389–411. doi: 10.18632/aging.205132 (PMC10637829; doi:10.18632/aging.205132)
Supplement: Supplementary Table 1 [file aging-15-205132-s002.pdf]

SUPPLEMENTARY TABLE

Supplementary Table 1. The list of ICDRGs.

IL17RA
IL1R1
PIK3CA
CD4
IFNG
PRF1
CXCR3
CD8A
CD8B
P2RX7
NLRP3
IL10
TLR4
ENTPD1
ATG5
IFNB1
IL6
EIF2AK3
IL17A
LY96
FOXP3
HMGB1
HSP90AA1
BAX
PDIA3
CALR
CASP8
MYD88
IFNGR1
CASP1
IL1B
TNF
NT5E
